# Impact of high-power short-duration atrial fibrillation ablation technique on the incidence of silent cerebral embolism: a prospective randomized controlled study

**DOI:** 10.1186/s12916-023-03180-3

**Published:** 2023-11-23

**Authors:** Wei-Jie Chen, Chun-Xia Gan, Yang-Wei Cai, Yang-Yang Liu, Pei-Lin Xiao, Li-Li Zou, Qing-Song Xiong, Fang Qin, Xie-Xin Tao, Ran Li, Hua-An Du, Zeng-Zhang Liu, Yue-Hui Yin, Zhi-Yu Ling

**Affiliations:** 1 Department of Cardiology, the Second Affiliated Hospital of Chongqing Medical University, No. 288, Tianwen Avenue, Chayuan, Nan’an District, Chongqing, 400060 China; 2 Department of Radiology, the Second Affiliated Hospital of Chongqing Medical University, Chongqing, China

**Keywords:** Atrial fibrillation, Catheter ablation, High-power short-duration, Silent cerebral embolism, High-resolution diffusion-weighted magnetic resonance imaging

## Abstract

**Background:**

High-power short-duration (HPSD) ablation strategy has emerged as a popular approach for treating atrial fibrillation (AF), with shorter ablation time. The utilized Smart Touch Surround Flow (STSF) catheter, with 56 holes around the electrode, lowers electrode-tissue temperature and thrombus risk. Thus, we conducted this prospective, randomized study to investigate if the HPSD strategy with STSF catheter in AF ablation procedures reduces the silent cerebral embolism (SCE) risk compared to the conventional approach with the Smart Touch (ST) catheter.

**Methods:**

From June 2020 to September 2021, 100 AF patients were randomized 1:1 to the HPSD group using the STSF catheter (power set at 50 W) or the conventional group using the ST catheter (power set at 30 to 35 W). Pulmonary vein isolation was performed in all patients, with additional lesions at operator’s discretion. High-resolution cerebral diffusion-weighted magnetic resonance imaging (hDWI) with slice thickness of 1 mm was performed before and 24–72 h after ablation. The incidence of new periprocedural SCE was defined as the primary outcome. Cognitive performance was assessed using the Montreal Cognitive Assessment (MoCA) test.

**Results:**

All enrolled AF patients (median age 63, 60% male, 59% paroxysmal AF) underwent successful ablation. Post-procedural hDWI identified 106 lesions in 42 enrolled patients (42%), with 55 lesions in 22 patients (44%) in the HPSD group and 51 lesions in 20 patients (40%) in the conventional group (*p* = 0.685). No significant differences were observed between two groups regarding the average number of lesions (*p* = 0.751), maximum lesion diameter (*p* = 0.405), and total lesion volume per patient (*p* = 0.669). Persistent AF and CHA_2_DS_2_-VASc score were identified as SCE determinants during AF ablation procedure by multivariable regression analysis. No significant differences in MoCA scores were observed between patients with SCE and those without, both immediately post-procedure (*p* = 0.572) and at the 3-month follow-up (*p* = 0.743).

**Conclusions:**

Involving a small sample size of 100 AF patients, this study reveals a similar incidence of SCE in AF ablation procedures, comparing the HPSD strategy using the STSF catheter to the conventional approach with the ST catheter.

**Trial registration:**

Clinicaltrials.gov: NCT04408716.

**Graphical Abstract:**

AF = Atrial fibrillation, DWI = Diffusion-weighted magnetic resonance imaging, HPSD = High-power short-duration, ST = Smart Touch, STSF = Smart Touch Surround Flow.

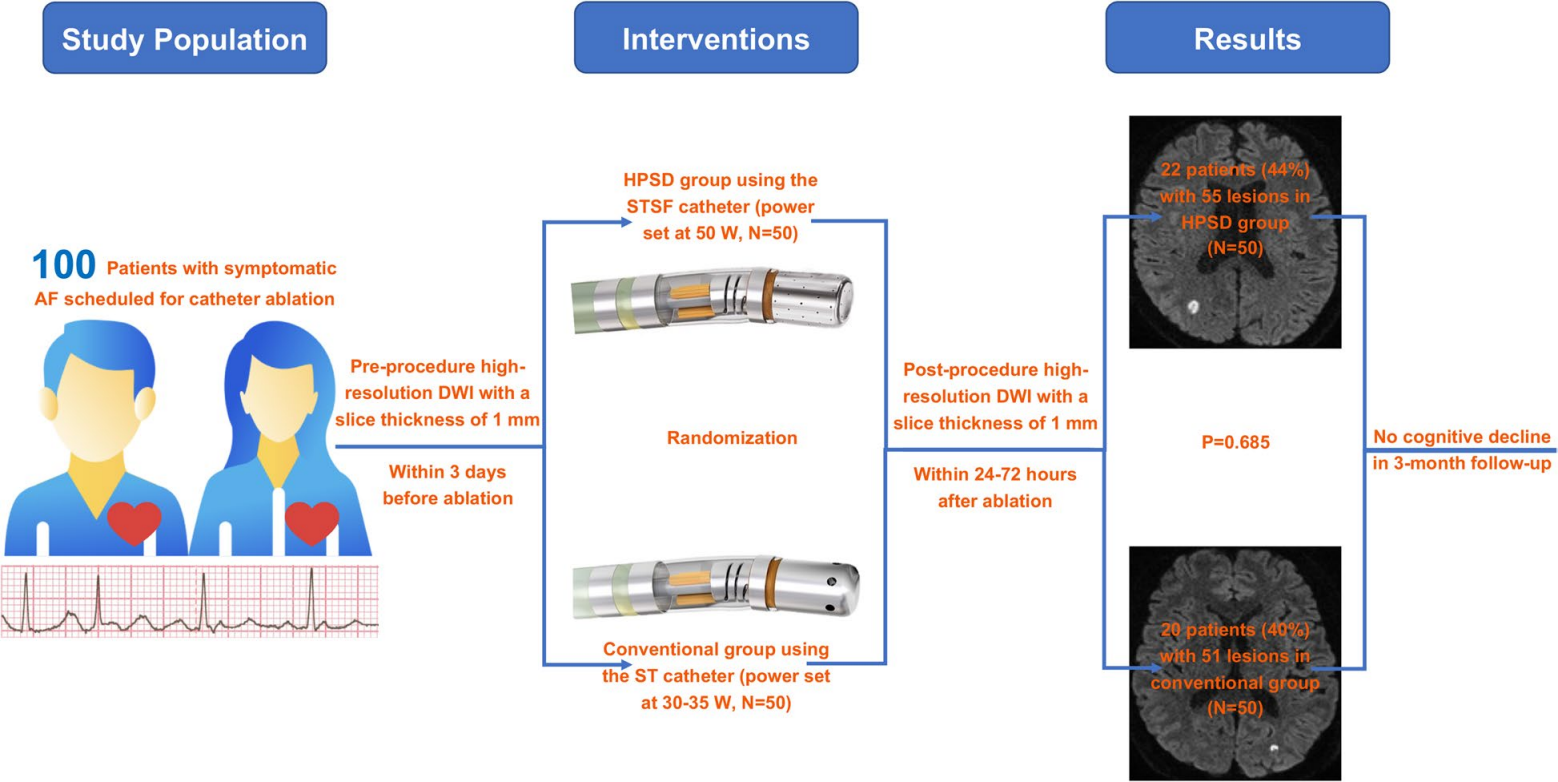

**Supplementary Information:**

The online version contains supplementary material available at 10.1186/s12916-023-03180-3.

## Background

Catheter ablation to achieve pulmonary vein isolation (PVI) has emerged as a guideline-recommended treatment option for patients with symptomatic atrial fibrillation (AF) [1, 2]. The efficacy and safety of the procedure have been repeatedly demonstrated in numerous studies over the past two decades. However, catheter ablation of atrial fibrillation is still associated with considerable risk of a series of complications. Cerebral embolic events are one of the common and harmful complications associated with AF ablation. The risk of symptomatic cerebral embolism is less than 1% during AF ablation procedure [3]; nevertheless, the incidence of silent cerebral embolism (SCE) detected by cerebral magnetic resonance imaging (MRI) is reported to be up to 67.3% [4]. Meanwhile, studies have also shown an association between SCE and the likelihood of cognitive impairment [5, 6]. Hence, the risk of periprocedural SCE during AF ablation, even if asymptomatic, should not be ignored.

In recent years, the high-power short-duration (HPSD) ablation strategy has been applied in the treatment of AF to produce the continuous, transmural lesions and limit collateral damages [7–12]. Previous studies have indicated that the application of HPSD strategy in AF ablation procedure is associated with shorter procedure time and ablation duration, higher first-pass PVI, reduced risk of acute and chronic PV reconnection, and lower risk of AF recurrence after a single ablation procedure [7–13]. The potential safety of the HPSD strategy in AF ablation has also been demonstrated by published studies [7, 12, 14–16]. However, as a common complication, the impact of the HPSD strategy on the incidence of SCE during AF ablation procedure has not been systematically investigated. The novel Smart Touch Surround Flow (STSF) catheter, which is designed with 56 small bore holes homogeneously distributed around the entire electrode surface to lower the electrode-tissue interface temperature, increase the delivery of radiofrequency (RF) energy to the tissue, and reduce the risk of thrombus formation [17, 18], has been widely used in the HPSD ablation procedure [8–12, 16]. Additionally, the total RF energy delivery time is shortened during AF ablation procedure using the HPSD strategy [7–13]. Therefore, we hypothesized that the application of the HPSD strategy using the STSF catheter in AF ablation procedures would significantly reduce the risk of periprocedural SCE, in contrast to the conventional AF ablation approach using the Smart Touch (ST) catheter with a power range of 30–35 W. To test this hypothesis, we conducted this prospective, randomized, and controlled study aiming to compare the cerebral safety of AF ablation procedures using the HPSD strategy with the STSF catheter versus the conventional approach with the ST catheter, with SCE detected by high-resolution brain diffusion-weighted magnetic resonance imaging (hDWI) (Reduce-IT Study; NCT number: NCT04408716).

## Methods

### Study design

This study was designed as a single-center, prospective, single-blind, randomized, controlled clinical trial to evaluate the periprocedural incidence of SCE in AF patients undergoing catheter ablation with the HPSD strategy versus the conventional approach. From June 2020 to September 2021, 100 AF patients undergoing their first catheter ablation procedure were enrolled in the study. Enrolled patients were randomly assigned to undergo catheter ablation with the HPSD strategy (HPSD group) or the conventional approach (Conventional group) using a randomization envelope. Patients in the HPSD group underwent point-by-point circumferential pulmonary vein ablation with the HPSD strategy (RF energy was set up at a power of 50 W, temperature of 43 °C, contact force of 5–20 g, and flow rate of 20 mL/min) using the novel STSF catheter. Patients in the conventional group underwent point-by-point circumferential pulmonary vein ablation with conventional settings (RF energy was set up at a power of 30 to 35 W, temperature of 43 °C, contact force of 5–20 g, and flow rate of 17 to 30 mL/min) using the ST catheter. The periprocedural incidence of SCE was determined by brain hDWI, which was performed 3 days before the procedure and re-evaluated 24–72 h after ablation. The study was approved by the ethics committee of the Second Affiliated Hospital of Chongqing Medical University and adhered to the tenets of the Declaration of Helsinki. Written informed consent was obtained from all enrolled patients.

### Study population

Eligible patients are those who (1) have electrocardiographically documented, symptomatic AF, (2) are scheduled to undergo their first catheter ablation procedure for AF, (3) are between the ages of 18 and 80 years, and (4) are willing and able to provide informed consent. Patients are excluded if they (1) have moderate to severe valvular heart disease, (2) are contraindicated for anticoagulation therapy, (3) are contraindicated for hDWI, (4) have had an ischemic stroke within 6 months prior to the consent date, (5) have had an acute coronary syndrome within 3 months prior to the consent date, (6) have ever had a left atrial appendage occlusion device or septal occlusion device, (7) have a significantly enlarged left atrium (left atrial diameter ≥ 55 mm), (8) have conditions that prevent their participation in the cognitive assessment, (9) be a female who is pregnant, breastfeeding, or planning to become pregnant during the study, (10) be concurrently enrolled in another study, and (11) be unwilling or unable to comply fully with the study protocol.

### Preprocedural managements

All enrolled patients received at least 3 weeks anticoagulation therapy based on the recommendation of guideline [1]. Transesophageal echocardiography was performed in all patients prior to the procedure to rule out intracardiac thrombus. To reduce the risk of bleeding events during the procedure, oral anticoagulants were paused on the morning of the ablation day and resumed after the procedure. Antiarrhythmic drugs were discontinued for at least 5 half-lives.

### Ablation procedure

Ablation procedures were performed under conscious sedation with continuous infusion of fentanyl. Under local anesthesia, after successful puncture of the bilateral femoral veins, a loading dose of 120 U/kg heparin was administered to achieve an activated clotting time (ACT) ≥ 300 S while ablation energy was delivered. A 6F diagnostic decapolar catheter (MicroPort EP, Shanghai, China) was positioned in the coronary sinus via the left femoral vein. After two 8.5F Swartz SL1 sheaths were advanced via the right femoral vein, sequential double transseptal punctures were performed under fluoroscopic guidance. The transseptal sheaths were continuously flushed with heparinized saline throughout the ablation procedure. A circular decapolar catheter (MicroPort EP, Shanghai, China) was inserted into the left atrium through a transseptal sheath to record the pulmonary vein ostia potential. A 3.5 mm open-irrigated ablation catheter (the STSF catheter for patients in the HPSD group, and the ST catheter for patients in the conventional group; Biosense-Webster, Diamond Bar, CA) was advanced through another transseptal sheath into the left atrium for ablation therapy. The three-dimensional electro-anatomic reconstruction of the left atrium (including atrium, pulmonary veins, left atrial appendage, mitral valve annulus) was performed using the irrigated ablation catheter under the guidance of the CARTO3 mapping system (CARTO 3 V6, Biosense-Webster, Diamond Bar, CA). Wide-area circumferential PVI was performed point-by-point under the guidance of the CARTO3 mapping system with an automated ablation lesion tagging program (VisiTag™ Module, Biosense-Webster, Diamond Bar, CA). The VisiTag criteria were set as follows: range of catheter motion ≤ 2.5 mm for 3S and contact force ≥ 5 g for more than 40% of the time. For patients in the HPSD group, RF energy was adjusted to 50 W, temperature 43 °C, contact force 5–20 g, and flow rate 20 mL/min using the novel STSF catheter, while for patients in the conventional group, RF energy was adjusted to 30–35 W, temperature 43 °C, contact force 5–20 g, and flow rate 17–30 mL/min using the ST catheter. For all enrolled patients, the target ablation index was set at 500 on the anterior wall and 350 on the posterior wall of the left atrium. Circumferential PVI was performed in all enrolled patients. Additional ablation lesions were performed at the discretion of the operator. If AF persisted after ablation, electrical cardioversion was performed to restore the sinus rhythm. With the mandatory administration of additional heparin, ACT was monitored every 30 min to maintain the target of 280–350 S throughout the ablation procedure. Anticoagulation therapy was resumed 4–6 h after the procedure when pericardial effusion and bleeding events were excluded by bedside echocardiography with ACT < 180 S.

### High-resolution diffusion-weighted magnetic resonance imaging

To explore the periprocedural incidence of SCE, 3.0-T scanner-based cerebral MRI (Magnetom Prisma, Siemens, Erlangen, Germany) was performed within 3 days before ablation and re-evaluated within 24–72 h after ablation. The imaging protocol for cerebral MRI consisted of a T2-weighted axial fluid-attenuated inversion recovery sequence and a diffusion-weighted magnetic resonance imaging (DWI) sequence. Apparent diffusion coefficient (ADC) maps were also generated for each DWI sequence. To precisely depict the periprocedural incidence of SCE, high-resolution cerebral magnetic resonance imaging was applied to the DWI and ADC sequences. The thickness of each slice was set to 1 mm, and the total number of slices was set to 100. Acute SCE was defined as a new hyperintense lesion on the DWI sequence with a corresponding hypointense area on the matched ADC sequence of the post-procedural brain MRI. The total number, size, volume, and location of SCE in patients with acute hDWI-detected SCE were analyzed by two experienced radiologists who were blinded to patient clinical status using 3D Slicer software [19]. Acute SCE was classified into three categories based on the size: small (< 3 mm in diameter), medium (3 to 10 mm in diameter), and large (≥ 10 mm in diameter). Disagreements between radiologists were resolved by discussion or consultation with a third radiologist.

### Cognitive assessments

To evaluate the potential impact of periprocedural SCE on cognition, the Montreal Cognitive Assessment (MoCA) test was administered to all enrolled patients on the day before ablation, 24–72 h after ablation, and at the 3-month follow-up. The MoCA test consists of 30 items assessing visuospatial ability, executive function, attention, short-term memory, concentration and working memory, language, and orientation to time and place. Patients can earn up to 30 points on the MoCA test, with higher scores indicating better cognitive function.

### Follow-up and outcomes

After discharge from the hospital, all enrolled patients returned to the hospital for scheduled follow-up visits at 3 and 6 months. Scheduled follow-up included clinical assessments, MoCA testing, and 24-h Holter monitoring. Patients received anticoagulation therapy for at least 3 months after the procedure, with the anticoagulation strategy determined by their CHA_2_DS_2_-VASc score at the 3-month follow-up. Antiarrhythmic drugs were prescribed during the first 3 months to reduce the risk of early recurrence, but were discontinued if the sinus rhythm persisted after 3 months. The primary outcome was the incidence of new SCE detected by post-procedural hDWI within the 24–72 h after ablation. The secondary outcomes were the safety endpoints during the procedure and at the 3-month follow-up, including cognitive impairment as assessed by the MoCA test and the overall complication rate.

### Statistical analysis

As detected by hDWI at a thickness of 1 mm, the reported incidence of SCE in AF patients undergoing conventional catheter ablation was as high as 67.3% [4]. To hypothesize the incidence of SCE in the HPSD group detected by hDWI, we conducted a preliminary study involving 16 AF patients who underwent catheter ablation using the HPSD strategy with the STSF catheter. The results showed that 5 out of the 16 patients (31.3%) suffered from acute SCE as detected by hDWI. Based on the reported incidence of SCE in conventional group as high as 67.3% and our preliminary findings, we assumed that the incidence of SCE would be 60% in the conventional group and 30% in the HPSD group. Upon conducting a sample size calculation and accounting for a potential 15% drop-out rate due to patients’ reluctance to undergo repeated hDWI post-ablation, at least 92 patients were required to achieve 80% power in detecting SCE differences between the two groups with an *α* level of 0.05. Consequently, the final sample size for this study was set at 100, with 50 patients allocated to each group. Randomization was performed using the online version of Research Randomizer [20]. A physician who was not involved in this study distributed the randomization results in sealed, opaque envelopes. After the patient was sterilized in the cath lab, a nurse was instructed to open the enclosed envelope, and the allocated ablation strategy was performed. Normality of variable distribution was assessed using the Shapiro–Wilk test. Continuous variables with normal distribution were expressed as mean ± standard deviation and analyzed using the Student’s *t*-test. Non-normally distributed continuous variables were presented as median with interquartile range (IQR) and compared using the Mann–Whitney *U* test. Categorical variables were reported as numbers and percentages, and the chi-square test or Fisher’s exact test was used for comparisons. Clinical characteristics and procedural parameters were also compared between SCE and non-SCE patients. The variables with *p* < 0.1 between SCE patients and non-SCE patients, as well as other clinically relevant variables such as age, persistent AF, CHA_2_DS_2_-VASc score, cardioversion, left atrial diameter, ablation strategy with PVI plus, initial ACT before energy delivery, mean ACT during the procedure, procedure time, radiofrequency time, and irrigation volume, were first included in the univariable logistic regression analysis. Furthermore, variables with *p* < 0.1 in the univariable analysis were selected for backward stepwise multivariable logistic regression analysis to investigate the predictors of SCE during the AF ablation procedure. However, even though the *p* value was less than 0.1 in the univariable analysis, the variables included in the CHA_2_DS_2_-VASc score were not repeated in the multivariable logistic regression analysis to avoid the potential interactions between the variables and the CHA_2_DS_2_-VASc score. The linear relationship between each continuous independent variable and the logit-transformed dependent variable was determined by using the Box–Tidwell test. A Bonferroni correction based on all the terms in the model was applied to assess the assumption of linearity. Meanwhile, the overall measure of discrimination of the model was determined by the AUC of the ROC curve. All tests were two-sided, and a *p* value of 0.05 was used to determine the statistical significance. All the statistical evaluations were performed using the SPSS 28.0.

## Results

### Patients characteristics

**Fig. 1:**
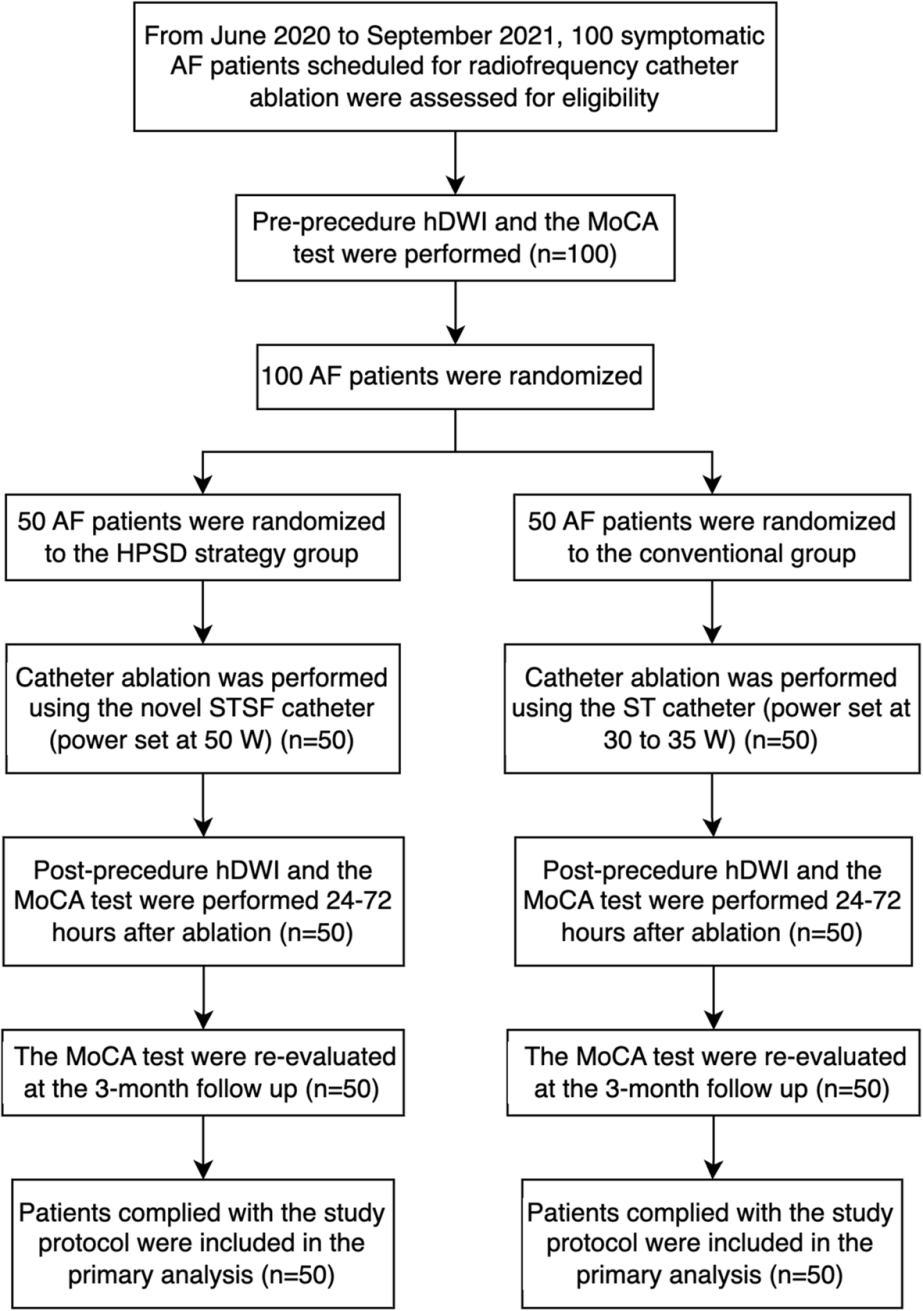
Flowchart of the present study. AF atrial fibrillation, hDWI high-resolution diffusion-weighted magnetic resonance imaging, HPSD high-power short-duration, MoCA Montreal Cognitive Assessment, ST Smart Touch, STSF Smart Touch Surround Flow

The flowchart of this study is depicted in Fig. 1, while Table 1 summarizes the baseline characteristics of the patients in both the HPSD and conventional groups. The median age of patients in both the HPSD group and conventional group was 63 years old (IQR 52–70 for the HPSD group and 55–70 for the conventional group). There were 27 (54%) male patients in the HPSD group and 33 (66%) male patients in the conventional group. The median CHA_2_DS_2_-VASc score was 1.5 points (IQR 1–3) in the HPSD group and 2 points (IQR 1–3) in the conventional group. In the HPSD group, 33 patients (66%) had paroxysmal AF, while the remaining 17 patients (34%) had persistent AF. In the conventional group, 26 patients (52%) had paroxysmal AF and 24 patients (48%) had persistent AF. The median left ventricular ejection fraction (LVEF) was 66% in both the HPSD group (IQR 61.7–71.0) and the conventional group (63.0–72.0). Hypertension was the most common comorbidity in both the HPSD group (44%) and the conventional group (50%), followed by hyperlipidemia (22% in the HPSD group, 26% in the conventional group), coronary artery disease (12% in the HPSD group, 34% in the conventional group), diabetes mellitus (12% in the HPSD group, 14% in the conventional group), stroke or transient ischemic attack (TIA) (14% in both groups), and heart failure (4% in both groups). The median NT-proBNP level was 226.3 pg/ml (IQR 74.9–881.6) in the HPSD group and 421.3 pg/ml (IQR 125.4–922.3) in the conventional group. Additionally, up to 98% of the enrolled patients took NOACs for anticoagulation therapy before ablation procedure, while only one patient in the HPSD group and one in the conventional group took warfarin for anticoagulation therapy (Additional file 1: Table S1). After the ablation procedure, one patient in the HPSD group switched from warfarin to rivaroxaban for anticoagulation therapy (Additional file 1: Table S1).

**Table 1:** Baseline characteristics of enrolled patients

**Variables**	**HPSD group** **(*n* = 50)**	**Conventional** **group** **(*n* = 50)**
**Age (years, IQR)**	63 (52–70)	63 (55–70)
**Male (*n*, %)**	27 (54%)	33 (66%)
**BMI (kg/m**^**2**^**, IQR)**	24.2 (22.0–26.4)	24.1 (22.2–26.3)
**Smoking (*n*, %)**	16 (32%)	21 (42%)
**Drinking (*n*, %)**	11 (22%)	19 (38%)
**CHA**_**2**_**DS**_**2**_**-VASc score (points, IQR)**	1.5 (1–3)	2.0 (1–3)
**MoCA score (points, IQR)**	26.0 (22.0–27.3)	25.5 (22.0–28.0)
**AF classification**		
Paroxysmal (*n*, %)	33 (66%)	26 (52%)
Persistent (*n*, %)	17 (34%)	24 (48%)
**Echocardiography**		
LAD (mm, mean ± SD)	37.5 ± 4.0	38.9 ± 5.3
LVEF (%, IQR)	66.0 (61.7–71.0)	66.0 (63.0–72.0)
**Comorbidities**		
Hypertension (*n*, %)	22 (44%)	25 (50%)
Coronary artery disease (*n*, %)	6 (12%)	17 (34%)
Diabetes mellitus (*n*, %)	6 (12%)	7 (14%)
Heart failure (*n*, %)	2 (4%)	2 (4%)
Stroke/TIA (including lacunar infarct, *n*, %)	7 (14%)	7 (14%)
Hyperlipidemia (*n*, %)	11 (22%)	13 (26%)
**Laboratory test**		
NT-proBNP level (pg/ml, IQR)	226.3 (74.9–881.6)	421.3 (125.4–922.3)
Creatinine (mmol/L, IQR)	70.1 (62.0–84.7)	72.2 (61.8–86.5)

*AF* atrial fibrillation, *BMI* body mass index, *HPSD* high-power short-duration, *IQR* interquartile range, *LAD* left atrial diameter, *LVEF* left ventricular ejection fraction, *MoCA* Montreal Cognitive Assessment, *TIA* transient ischemic attack

### Procedure results

Procedure information is displayed in Table 2. All enrolled patients underwent successful wide-area circumferential PVI with verification of bidirectional block. Additional ablation lesions were performed in 22 patients (44%) in the HPSD group and 30 patients (60%) in the conventional group (*p* = 0.109). There were no significant differences in the detailed additional ablation strategies between the two groups. The first-pass isolation rate for the right-sided pulmonary veins was 64% in the HPSD group and 68% in the conventional group (*p* = 0.673), while the first-pass PVI for the left-sided pulmonary veins was 82% in the HPSD group and 80% in the conventional group (*p* = 0.799). Eleven patients (22%) in the HPSD group and 19 patients (39%) in the conventional group received electrical cardioversion during the procedure (*p* = 0.081). In the HPSD group, the procedure time, radiofrequency time, and irrigation volume were significantly reduced compared to the conventional group (*p* = 0.012, *p* < 0.001, *p* < 0.001, respectively). No significant difference was found in fluoroscopy time between the two groups (*p* = 0.560). Regarding procedural anticoagulation management, the median ACT before energy delivery was 292.5 S in the HPSD group (IQR 273.0–329.5) and 299.0 S in the conventional group (IQR 259.0–333.5) (*p* = 0.634). Meanwhile, the median mean ACT during the procedure was 285.0 S in the HPSD group (IQR 274.3–305.5) and 295.5 S in the conventional group (IQR 282.0–313.0) (*p* = 0.094). There were also no significant differences between the two groups in maximum ACT (*p* = 0.287), minimum ACT (*p* = 0.101), loading dose of heparin (*p* = 0.131), and total dose of heparin (*p* = 0.470). Regarding procedural safety events, three patients (6%) in the HPSD group and one patient (2%) in the conventional group experienced steam pop during the procedure (*p* = 0.617), whereas one patient (2%) in the HPSD group and two patients (4%) in the conventional group experienced pericardial effusion (*p* = 0.99), which was successfully controlled by pericardial aspiration.

**Table 2:** Comparisons of procedural data between the HPSD group and conventional group

**Variables**	**HPSD group (*n* = 50)**	**Conventional group (*n* = 50)**	***P*** **value**
**Procedure time (min, IQR)**	181.0 (150.0–222.5)	213.0 (180.0–242.5)	0.012^*^
**Radiofrequency time (min, IQR)**	29.5 (24.7–43.0)	47.0 (40.8–53.0)	< 0.001^*^
**Irrigation volume (ml, IQR)**	666.0 (544.8–950.0)	984.5 (869.0–1137.5)	< 0.001^*^
**Fluoroscopy time (min, IQR)**	6.0 (4.0–9.0)	5.5 (3.0–9.0)	0.560^*^
**Cardioversion (*n*, %)**	11 (22%)	19 (38%)	0.081^†^
**First pass PVI**			
RPV (*n*, %)	32 (64%)	34 (68%)	0.673^†^
LPV (*n*, %)	41 (82%)	40 (80%)	0.799^†^
**Ablation strategy**			
PVI only (*n*, %)	28 (56%)	20 (40%)	0.109^†^
PVI plus (*n*, %)	22 (44%)	30 (60%)	
Roof line (*n*, %)	17 (34%)	23 (46%)	0.221^†^
Floor line (*n*, %)	12 (24%)	18 (36%)	0.190^†^
Mitral isthmus line (*n*, %)	8 (16%)	11 (22%)	0.444^†^
Cavotricuspid isthmus line (*n*, %)	9 (18%)	11 (22%)	0.617^†^
SVC isolation (*n*, %)	4 (8%)	4 (8%)	0.99^‡^
Substrate modification (*n*, %)	3 (6%)	4 (8%)	0.99^‡^
**Procedural anticoagulation management**			
Loading dose of heparin (IU, IQR)	7750 (7000–8000)	8000 (7500–8625)	0.131^*^
Total dose of heparin (IU, IQR)	8500 (7500–11500)	9000 (8000–11250)	0.470^*^
ACT before energy delivery (S, IQR)	292.5 (273.0–329.5)	299.0 (259.0–333.5)	0.634^*^
Mean ACT during procedure (S, IQR)	285.0 (274.3–305.5)	295.5 (282.0–313.0)	0.094^*^
Maximum ACT during procedure (S, IQR)	304.0 (290.3–333.5)	317.5 (298.0–338.5)	0.287^*^
Minimum ACT during procedure (S, IQR)	270.0 (246.0–285.0)	279.0 (250.3–298.5)	0.101^*^
**Steam pop (*n*, %)**	3 (6%)	1 (2%)	0.617^‡^
**Pericardial effusion (*n*, %)**	1 (2%)	2 (4%)	0.99^‡^

*ACT* activated clotting time, *HPSD* high-power short-duration, *IQR* interquartile range, *LPV* left-sided pulmonary vein, *PVI* pulmonary vein isolation, *RPV* right-sided pulmonary vein, *SVC* superior vena cava

^*^Mann–Whitney *U* test

^†^Chi-square test

^‡^Fisher’s exact test

### Cerebral high-resolution magnetic resonance imaging findings

**Fig. 2:**
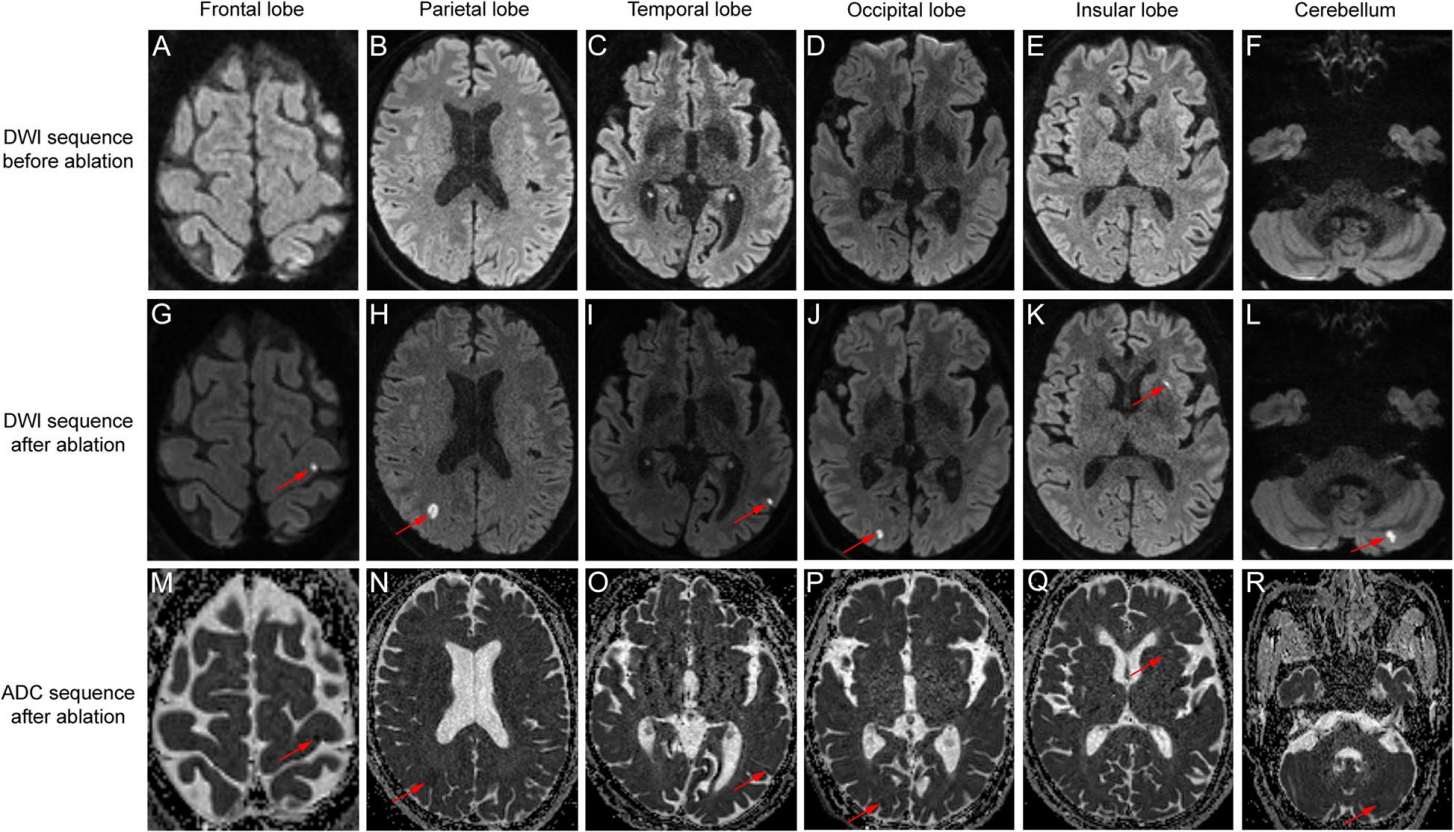
The representative acute lesions identified by high-resolution diffusion-weighted magnetic resonance imaging. DWI Diffusion-weighted magnetic resonance imaging, ADC apparent diffusion coefficient magnetic resonance imaging

All enrolled patients underwent pre-procedural and post-procedural 3.0 T cerebral hDWI, and no patients were eliminated due to MRI data missing. On pre-procedural cerebral hDWI, lacunar infarction was detected in seven patients (14%) in the HPSD group and six patients (12%) in the conventional group (*p* = 0.766), while one patient in the conventional group had obsolete cerebral infarct lesions (Table 1). The findings of cerebral hDWI are summarized in Table 3. Post-procedural hDWI identified a total of 106 lesions in 42 enrolled patients (42%), with 55 lesions in 22 patients (44%) in the HPSD group and 51 lesions in 20 patients (40%) in the conventional group (*p* = 0.685). The representative acute lesions identified by hDWI are displayed in Fig. 2. None of the patients with lesions detected by hDWI exhibited associated symptoms. In both the HPSD and conventional groups, nearly half of the lesions had a diameter of less than 3 mm, and the other half had a diameter between 3 and 10 mm, with only one lesion in a patient in the conventional group having a diameter greater than 10 mm. No differences were found between the two groups in lesions classification based on diameter (*p* = 0.637). The median average number of lesions was 2 in both groups (IQR 1.0–3.25 for the HPSD group and 1.0–3.0 for the conventional group, *p* = 0.751). The median maximum lesion diameter was 4.96 mm in the HPSD group (IQR 3.57–6.36) and 4.14 mm in the conventional group (IQR 3.19–5.67), with no statistical difference between the two groups (*p* = 0.406). In addition, there were also no significant differences observed between the two groups in terms of the median total lesion volume per patient (*p* = 0.669), with 46.8 mm^3^ in the HPSD group (IQR 24.1–148.3) and 43.3 mm^3^ in the conventional group (IQR 22.7–104.1). Furthermore, the cerebral location of the 106 identified SCE is summarized in Table 4, which shows that 92% of the lesions were distributed in the cortical and subcortical areas of the telencephalon, with the remaining 8% being discovered in the cerebellum.

**Table 3:** Comparisons of SCE between the HPSD group and conventional group

**Variables**	**HPSD group (*n* = 22)**	**Conventional group (*n* = 20)**	***P*** **value**
**Patients with SCE (*n*, %)**	22 (44%)	20 (40%)	0.685^†^
**Average number of lesions (*n*, IQR)**	2.0 (1.0–3.25)	2.0 (1.0–3.0)	0.751^*^
**Maximum lesion diameter (mm, IQR)**	4.96 (3.57–6.36)	4.14 (3.19–5.67)	0.406^*^
**Classification of lesions**			
Small lesions (diameter < 3 mm) (*n*, %)	26/55 (47.3%)	27/51 (52.9%)	0.637^*^
Medium lesions (3 mm ≤ diameter < 10 mm) (*n*, %)	29/55 (52.7%)	23/51 (45.1%)	
Large lesions (diameter ≥ 10 mm) (*n*, %)	0	1/51 (2.0%)	
**Total lesion volume per patient (mm**^**3**^**, IQR)**	46.8 (24.1–148.3)	43.3 (22.7–104.1)	0.669^*^

*HPSD* high-power short-duration, *IQR* interquartile range, *SCE* silent cerebral embolism

^*^Mann–Whitney *U* test

^†^Chi-square test

**Table 4:** The cerebral location of SCE in 42 positive patients

**Location**	**Frontal lobe**	**Parietal lobe**	**Temporal lobe**	**Insular lobe**	**Occipital lobe**	**Cerebellum**
**Left (*n*)**	23	12	5	1	7	3
**Right (*n*)**	22	14	3	1	10	5
**Total (*n*, %)**	45 (42.5%)	26 (24.5%)	8 (7.5%)	2 (1.9%)	17 (16.1%)	8 (7.5%)

*SCE* silent cerebral embolism

### Clinical and procedural features of SCE patients and non-SCE patients

The clinical characteristics of SCE patients and non-SCE patients were analyzed and reported in Table 5. The median age of SCE patients was 66 years old (IQR 55–75), whereas the median age of non-SCE patients was 61 years (IQR 51–68) (*p* = 0.038). The median CHA_2_DS_2_-VASc score was 2 points in SCE patients (IQR 1–3) and 1 point in non-SCE patients (IQR 1–3) (*p* = 0.002). Regarding AF classification, 40 non-SCE patients (69%) and 19 SCE patients (45.2%) had paroxysmal AF (*p* = 0.017), whereas 18 non-SCE patients (31%) and 23 SCE patients (54.8%) had persistent AF (*p* = 0.020). Left atrial diameter was slightly greater in SCE patients than that in non-SCE patients (39.3 ± 4.9 vs. 37.4 ± 4.5, *p* = 0.047). No significant differences were found in LVEF between SCE and non-SCE patients (*p* = 0.376). Hypertension and coronary artery disease were significantly more common in SCE patients than in non-SCE patients (*p* < 0.001, *p* = 0.037, respectively). There were no discernible differences between SCE patients and non-SCE patients for other comorbidities, such as diabetes (*p* = 0.745), heart failure (*p* = 0.137), stroke or transient ischemic attack (*p* = 0.944), and hyperlipidemia (*p* = 0.144). No significant differences were found between SCE patients and non-SCE patients in the NT-proBNP and creatinine levels (*p* = 0.230, *p* = 0.992, respectively).

**Table 5:** Comparisons of clinical characteristics between SCE and non-SCE patients

**Variables**	**SCE patients (*n* = 42)**	**Non-SCE patients (*n* = 58)**	***P*** **value**
**Age (years, IQR)**	66 (55–75)	61 (51–68)	0.038^*^
**Male (*n*, %)**	26 (61.9%)	34 (58.6%)	0.741^†^
**CHA**_**2**_**DS**_**2**_**-VASc score (points, IQR)**	2 (1–3)	1 (1–3)	0.002^*^
**MoCA score**			
Baseline (points, IQR)	26.5 (21.8–28.0)	26.0 (22.0–27.0)	0.888^*^
Post procedure (points, IQR)	26.0 (22.0–28.0)	26.0 (23.0–28.3)	0.572^*^
3 months (points, IQR)	26.0 (24.0–29.3)	27.5 (24.0–28.3)	0.743^*^
**Ablation strategy**			
HPSD strategy (*n*, %)	22 (52.4%)	28 (48.3%)	0.685^†^
Conventional approach (*n*,%)	20 (47.6%)	30 (51.7%)	
**AF classification**			
Paroxysmal (*n*, %)	19 (45.2%)	40 (69%)	0.017^†^
Persistent (*n*, %)	23 (54.8%)	18 (31%)	0.020^†^
**Echocardiography**			
LAD (mm, mean ± SD)	39.3 ± 4.9	37.4 ± 4.5	0.047^¶^
LVEF (%, IQR)	66.0 (63.8–71.3)	66.0 (61.8–71.3)	0.376^*^
**Comorbidities**			
Hypertension (*n*, %)	28 (66.7%)	19 (32.8%)	< 0.001^†^
Coronary artery disease (*n*, %)	14 (33.3%)	9 (15.5%)	0.037^†^
Diabetes mellitus (*n*, %)	6 (14.3%)	7 (12.1%)	0.745^†^
Heart failure (*n*, %)	0	4 (7.0%)	0.137^‡^
Stroke/TIA (*n*, %)	6 (14.3%)	8 (13.8%)	0.944^†^
Hyperlipidemia (*n*, %)	7 (16.7%)	17 (29.3%)	0.144^†^
**Laboratory test**			
Creatinine (mmol/L, IQR)	71.1 (64.3–85.6)	70.1 (61.8–86.0)	0.992^*^
NT-proBNP level (pg/ml, IQR)	415.3 (123.8–1008.1)	219.1 (107.0–766.3)	0.230^*^

*AF* atrial fibrillation, *HPSD* high-power short-duration, *IQR* interquartile range, *LAD* left atrial diameter, *LVEF* left ventricular ejection fraction, *MoCA* Montreal Cognitive Assessment, *SCE* silent cerebral embolism, *TIA* transient ischemic attack

^*^Mann–Whitney *U* test

^¶^Student’s t-test

^†^Chi-square test

^‡^Fisher’s exact test

The procedural parameters between SCE and non-SCE patients are summarized in Table 6. Ablation with PVI plus strategy was performed in 27 SCE patients (64.3%) and 25 non-SCE patients (43.1%), whereas PVI only strategy was performed in 15 SCE patients (35.7%) and 33 non-SCE patients (56.9%). The proportion of PVI plus strategy in SCE patients was significantly higher than that in non-SCE patients, whereas the PVI only approach was much more common in non-SCE patients than in SCE patients (*p* = 0.036). Electrical cardioversion was performed during the procedure in 17 SCE patients (40.3%) and 13 non-SCE patients (22.4%) (*p* = 0.052). No significant differences were found between the SCE patients and non-SCE patients in terms of procedure time (*p* = 0.122), RF time (*p* = 0.902), and irrigation fluid volume (*p* = 0.603). Regarding anticoagulation management, there were also no significant differences between the SCE patients and non-SCE patients in initial ACT before energy delivery (*p* = 0.572), mean ACT during the procedure (*p* = 0.227), loading and total heparin doses (*p* = 0.251, *p* = 0.253), and the maximum and the minimum ACT during the procedure (*p* = 0.149, *p* = 0.426). One SCE patient and three non-SCE patients experienced the steam pop (*p* = 0.637). All three pericardial effusion events occurred in non-SCE patients (*p* = 0.262).

**Table 6:** Comparisons of procedural parameters between SCE and non-SCE patients

**Variables**	**SCE patients (*n* = 42)**	**Non-SCE patients (*n* = 58)**	***P*** **value**
**PVI only (*n*, %)**	15 (35.7%)	33 (56.9%)	0.036^†^
**PVI plus (*n*, %)**	27 (64.3%)	25 (43.1%)	
**Cardioversion (*n*, %)**	17 (40.3%)	13 (22.4%)	0.052^†^
**Procedure time (min, IQR)**	210.0 (175.0–245.0)	190.0 (150.0–230.0)	0.122^†^
**Radiofrequency time (min, mean ± SD)**	40.6 ± 13.5	40.3 ± 11.9	0.902^¶^
**Irrigation volume (ml, mean ± SD)**	896.5 ± 268.1	866.9 ± 257.6	0.603^¶^
**Loading dose of heparin (IU, IQR)**	8000 (7375–8500)	8000 (7000–8500)	0.251^*^
**Total dose of heparin (IU, IQR)**	10,000 (8000–12,000)	8750 (8000–11,000)	0.253^*^
**ACT before energy delivery (S, IQR)**	292.0 (273.0–326.0)	299.5 (268.8–335.0)	0.572^*^
**Mean ACT during procedure (S, IQR)**	288.5 (266.8–303.0)	296.0 (279.8–311.3)	0.227^*^
**Maximum ACT during procedure (S, IQR)**	301.5 (288.0–330.3)	317.0 (298.0–336.3)	0.149^*^
**Minimum ACT during procedure (S, IQR)**	275.0 (245.3–285.3)	276.5 (254.5–291.8)	0.426^*^
**Steam pop (*n*, %)**	1 (2.4%)	3 (5.2%)	0.637^‡^
**Pericardial effusion (*n*, %)**	0	3 (5.2%)	0.262^‡^

*ACT* activated clotting time, *IQR* interquartile range, *PVI* pulmonary vein isolation, *SCE* silent cerebral embolism

^*^Mann–Whitney *U* test

^¶^Student’s *t*-test

^†^Chi-square test

^‡^Fisher's exact test

### Cognitive function of SCE patients and non-SCE patients

As shown in Table 5, the baseline MoCA score did not differ between SCE and non-SCE patients (*p* = 0.888). There were also no significant differences in MoCA scores between patients with SCE and those without SCE at the post-procedural (*p* = 0.572) and 3-month follow-up (*p* = 0.743) (Table 5). Meanwhile, no patient with SCE experienced significant cognitive impairment during the 3-month follow-up.

### Predictors of SCE during the AF ablation procedure

To investigate the potential predictors of SCE during the AF ablation procedure, univariable regression analysis was first performed (Table 7). Furthermore, backward stepwise multivariable logistic regression was performed by including the variables with *p* value less than 0.1 in the univariable analysis, including age, persistent AF, CHA_2_DS_2_-VASc score, cardioversion, coronary artery disease, left atrial diameter, ablation with PVI plus strategy, and procedure time. According to the results of multivariable regression analysis in Table 7, only persistent AF (OR, 2.689 [95% CI, 1.132–6.387], *p* = 0.025) and CHA_2_DS_2_-VASc score (OR, 1.568 [95% CI, 1.143–2.150], *p* = 0.005) were finally identified as the main risk factors for SCE occurrence during the AF ablation procedure. Meanwhile, the area under the ROC curve was 0.722 (95% CI, 0.622–0.822).

**Table 7:** Univariable and multivariable logistic regression analysis of periprocedural SCE

**Variables**	**Univariable regression analysis**			**Backward stepwise multivariable regression analysis**			
	**OR**	**95% CI**	***P* value**	**OR**	**95% CI**	***P* value**	**Elimination step**
**Age**	1.036	0.998–1.075	0.061	0.998	0.947–1.052	0.947	3
**Persistent AF**	2.690	1.180–6.131	0.019	2.689	1.132–6.387	0.025	In the equation
**CHA**_**2**_**DS**_**2**_**-VASc score**	1.570	1.154–2.136	0.004	1.568	1.143–2.150	0.005	In the equation
**Cardioversion**	2.354	0.984–5.630	0.054	1.031	0.273–3.901	0.964	2
**Coronary artery disease**	2.22	1.045–7.092	0.040	1.960	0.678–5.637	0.214	7
**Hypertension**^**a**^	4.105	1.765–9.547	0.001	——	——	——	——
**LAD (mm)**	1.092	1.000–1.194	0.051	0.970	0.859–1.095	0.621	5
**PVI plus**	2.376	1.049–5.382	0.038	1.271	0.403–4.012	0.682	4
**Initial ACT before energy delivery**	0.999	0.990–1.008	0.851	——	——	——	——
**Mean ACT during procedure**	0.999	0.987–1.011	0.841	——	——	——	——
**Procedure time**	1.007	0.999–1.014	0.090	1.003	0.994–1.012	0.491	6
**Radiofrequency time**	1.002	0.971–1.034	0.901	——	——	——	——
**Irrigation volume**	1.000	0.999–1.002	0.599	——	——	——	——

*ACT* activated clotting time, *AF* atrial fibrillation, *LAD* left atrial diameter, *PVI* pulmonary vein isolation, *SCE* silent cerebral embolism

^a^As hypertension was contained within the CHA_2_DS_2_-VASc score, even the *p* values were less than 0.1 in the univariable analysis; it was not repeatedly entered in the multivariable logistic regression analysis to avoid potential interactions between hypertension and the CHA_2_DS_2_-VASc score

## Discussion

### Main findings

Among published studies comparing the HPSD technique with the conventional approach, this is the first RCT using the incidence of periprocedural SCE as the primary outcome. The main findings are summarized as follows. (1) Despite detecting lesions using brain hDWI at a thickness of 1 mm, no significant differences were found in the incidence, average lesion number, maximum lesion diameter, and total lesion volume of SCE between patients in the HPSD group using the novel STSF catheter and the conventional group using the ST catheter. (2) Persistent AF and CHA_2_DS_2_-VASc score were identified as the main risk factors for the occurrence of SCE during the AF ablation procedure. (3) None of the patients with SCE showed cognitive impairment during the 3-month follow-up period.

Cerebral embolic lesions, reported up to 67.3% [4], are the most common complications associated with AF ablation procedure. By utilizing the STSF catheter, the HPSD ablation strategy has been indicated to be associated with shorter ablation time, greater first-pass PVI, lower risk of acute and chronic PV reconnection, and reduced risk of AF recurrence after a single ablation procedure, with reliable procedural safety [7–16]. However, the incidence of SCE or stroke during HPSD procedure has only been reported as a safety event in a few published studies [9, 10, 21–23]. Meanwhile, published studies have indicated an association between the occurrence of SCE and the likelihood of cognitive impairment [5, 6]. The question that, compared to the conventional approach using the ST catheter, whether the HPSD strategy using the STSF catheter would decrease the incidence of SCE and its potential impact on patients’ cognitive function has been repeatedly proposed by clinical doctors. Thus, to comprehensively assess the incidence of SCE during the HPSD procedure and its potential impact on patients’ cognitive function, we performed the present randomized study, with the primary outcome directly defined as the incidence of periprocedural SCE. Moreover, to depict the occurrence of SCE objectively and accurately, this study is the first application of the hDWI technique in clinical trials involving the HPSD ablation strategy. Finally, as shown in the present study, SCE events were identified in 22 patients (44%) in the HPSD group and in 20 patients (40%) in the conventional group, with no significant differences between the two groups. Furthermore, the detailed lesion characteristics such as maximum lesion diameter, average lesion number, and the total lesion volume were also thoroughly analyzed and compared, but no significant differences were found. Despite the fact that the procedure time and RF time were significantly shorter in the HPSD group, as well as the utilization of a novel 56-hole STSF catheter with a lower electrode–tissue interface temperature, the incidence of SCE was not effectively reduced in the HPSD group compared with the conventional group. Similar to the present study, Dr. Scaglione et al. [24] conducted a randomized pilot study 10 years ago to investigate the brain safety of open-irrigated catheters with different irrigation designs during AF ablation procedures, including the Thermocool Surround Flow 56-hole catheter and the Thermocool 6-hole catheter. The pilot study enrolled 80 patients with paroxysmal AF and randomized them 1:1 to the Thermocool Surround Flow group or the Thermocool group [24]. The authors also performed a cerebral MRI before and after the AF ablation procedure. Their results showed that two patients (5%) in the Thermocool Surround Flow group and three patients (7.5%) in the Thermocool group suffered from SCE, with no statistical difference between the two groups (*p* = 0.500) [24]. As the thickness of the cerebral MRI in the study by Dr. Scaglione et al. was 5 mm [24], the reported incidence of SCE in the study was only 6.25% (5/80). To accurately display the occurrence of SCE during the AF ablation procedure, the cerebral MRI slice thickness was established at 1 mm in the present study. As a result, the incidence of SCE in the present study was up to 42% (42/100), which is significantly higher than that in the study by Dr. Scaglione et al. Furthermore, the detailed lesion characteristics, including maximum lesion diameter, average lesion number, and total lesion volume, were analyzed and compared in the present study, but no significant differences were found between the two groups. Thus, by using the hDWI method and performing the thorough analysis, the results of the present study were much more accurate and reliable. Additionally, in the study by Dr. Scaglione et al. [24], the delivered power for both the Thermocool Surround Flow catheter and the Thermocool catheter was 30 W, increasing to 40 W only when atrial potentials failed to ablate. In contrast, the present study revealed the brain safety of the popular HPSD technique with the power setting at 50 W.

However, the results of recently published SHORT-AF study [25] indicated a trend towards a higher incidence of acute SCE with the HPSD strategy (10 out of 25, 40%) compared to the conventional approach (5 out of 30, 17%), without reaching statistical significance (*p* = 0.053). It is worth noting that the primary objective between the SHORT-AF study and the present study is completely different. The SHORT-AF study was designed as an RCT to test the hypothesis that HPSD resulted in a shorter procedure than SPSD without compromising efficacy or safety [25]. Thus, the primary outcome of the SHORT-AF study was defined as time to achieve PVI, while the incidence of acute SCE was just defined as one of the secondary safety endpoints [25]. In contrast, the present study is the first RCT in which the primary objective was to comprehensively assess the incidence of SCE during the HPSD procedure. Correspondingly, the primary outcome of the present study was the incidence of new SCE detected by post-procedural hDWI within the 24–72 h after ablation. Additionally, the aims and assumptions for sample size calculations between the two studies are also completely different. The sample size of 60 patients in the SHORT-AF study was determined to detect a 17-min difference in PVI time between the two groups, but not calculated to detect the difference in the incidence of acute SCE between the two groups [25]. Given that the conclusion about a trend toward a higher incidence of acute SCE for patients in the HPSD group was drawn from the secondary safety endpoints of the SHORT-AF study, the interpretation of this finding should be approached with caution and be verified in future randomized studies. In contrast, the sample size of 100 patients in the present Reduce-IT study was calculated to directly detect the difference in the incidence of acute SCE between the HPSD group and the conventional group. However, regarding the findings of these two studies, the revealed incidence of acute SCE for patients in the HPSD group appeared comparable, with 40% (10/25) in the SHORT-AF study [25] and 44% (22/50) in the present Reduce-IT study. Notably, despite utilizing a similar high-resolution MRI technique, the SHORT-AF study reported a 17% (5/30) incidence of acute SCE for its conventional group [25]. That is significantly lower than the 40% (20/50) in the present Reduce-IT study and the 67.3% (37/50) from a previously published study [4]. Based on above discussion, the reported trend of an increased risk of acute SCE with the HPSD strategy, coupled with the notably lower incidence of acute SCE for the conventional group in the SHORT-AF study, could be potentially attributed to its limited sample size. Additionally, in the SHORT-AF study, both the HPSD and conventional groups underwent AF ablation procedures using either the CARTO or EnSite system, each employing distinct catheter designs [25]. Such variations, compounded by the small sample size lacking statistical evaluation, intensified the heterogeneity and instability in the findings of secondary safety endpoints, such as the incidence of acute SCE. Therefore, the findings of the SHORT-AF study, regarding a trend towards a higher incidence of acute SCE with the HPSD strategy based on secondary endpoints, should be interpreted with caution and verified in future randomized studies. Interestingly, the present randomized Reduce-IT study seemingly just further investigated and addressed the concerns raised by the SHORT-AF study regarding the trend towards a higher incidence of acute SCE with the HPSD strategy. Furthermore, the detailed lesion characteristics such as maximum lesion diameter, average lesion number, and the total lesion volume were also thoroughly analyzed and compared in the present study, but still no significant differences were found.

Up to 42 enrolled patients (42%) had SCE in the present study. Although catheter ablation without anticoagulant interruption is recommended at a class I level [1, 2], the minimally interrupted oral anticoagulants approach with holding one to two doses of DOAC before AF ablation procedure is recommended as reasonable (class IIa) by the AF catheter ablation expert consensus [26] during the protocol design stage of the present study. Thus, the minimally interrupted oral anticoagulants approach was employed in the present study. We acknowledged that omitting the oral anticoagulants on the morning of the ablation day may have increased the occurrence risk of SCE [27]. However, a randomized study conducted in Japan [28], which enrolled 846 Asian patients, showed that both the uninterrupted and minimally interrupted protocols demonstrated a low risk of symptomatic thromboembolisms and a similar incidence of silent cerebral ischemic lesions. Meanwhile, the periprocedural anticoagulation management protocol is similar for all enrolled patients, regardless of whether they are in the HPSD group or the conventional group. Correspondingly, the risk of SCE from omitting anticoagulation on the ablation day was equal for patients in both groups. To investigate the risk factors for SCE during the AF ablation procedure, clinical characteristics and procedural parameters were compared between patients with and without SCE. Univariable and backward stepwise multivariable regression analyses were further performed. The results showed that persistent AF and CHA_2_DS_2_-VASc score were the main risk factors for SCE during the AF ablation procedure. We can easily find that the identified risk factors of persistent AF and CHA_2_DS_2_-VASc score were both the patient-level variables but not the procedure-related factors. As shown by the univariable regression analysis, procedural parameters of ablation with PVI plus strategy and procedure time were potential risk factors for SCE, but were excluded from the equation of multivariable regression analyses. These results indicate that the weight of the patient-related factors of persistent AF and CHA_2_DS_2_-VASc score in the occurrence of SCE during AF ablation procedure is substantially higher than that of ablation with PVI plus strategy and procedure time, although these two procedural parameters were also associated with the risk of SCE. However, the interpretation of this finding should be cautious. The previous study conducted in Chinese patients using the hDWI method already indicated that a procedural parameter of ACT > 283 S was an independent factor in lessening the risk of SCE during AF ablation procedures [4]. In the present study, the median ACT before energy delivery was up to 292.0 S in patients with SCE and 299.5 S in patients without SCE (*p* = 0.572), while the median mean ACT during the procedure was also up to 288.5 S in patients with SCE and 296.0 S in patients without SCE (*p* = 0.227). Thus, we propose that the effective management of procedural anticoagulation in the present study plays a critical role in reducing the risk of procedural parameters in the occurrence of SCE and should be considered as a prerequisite for interpreting the findings.

As previously discussed, patient-related characteristics of persistent AF and CHA_2_DS_2_-VASc score were subsequently identified as the most relevant risk factors for SCE during AF ablation procedure, after procedural anticoagulation management was improved to reduce the procedure-related risk of SCE. Previous non-randomized studies had also shown that the patient-related characteristics of persistent AF [29–31] and CHA_2_DS_2_-VASc score [32, 33] were the main predictors of SCE during AF ablation procedure. There’s no doubt that patients with a higher CHA_2_DS_2_-VASc score have a higher prevalence of cardiovascular risk factors and diseases, such as heart failure, hypertension, older age, diabetes, stroke, and vascular disease. Consequently, these patients are more likely to develop persistent AF [1, 34]. Meanwhile, patients with persistent AF and a higher CHA_2_DS_2_-VASc score are more likely to have a markedly enlarged left atrium as a result of the atrial remodeling process [1, 34, 35]. Left atrial contractility is notably diminished in patients with persistent AF and an enlarged left atrium [35, 36]. Accordingly, the blood flow velocity in the left atrium is also significantly reduced [35–37]. As a result, during the delivery of RF energy, there is a dramatic increase in the risk of thrombus and char formation, which are the crucial mechanisms contributing to the occurrence of SCE during AF ablation procedures [38–40]. Meanwhile, published study has indicated that most microemboli are gaseous in nature and are generated immediately after RF energy delivery [41]. As such, even with intensive procedural anticoagulation management, the risk of char formation, thrombus development, and gaseous microemboli resulting from RF energy delivery is unavoidable, especially in patients with persistent AF or a significantly enlarged left atrium. Additionally, in patients with persistent AF, the performance of PVI plus strategy and cardioversion during the ablation procedure also increases the risk of SCE, as these two factors were identified as risk factors by univariable analysis but were excluded from the equation of the multivariable regression analysis after the variables of persistent AF and CHA_2_DS_2_-VASc score were introduced. As shown in Table 5 of the present study, the incidence of SCE is 56.1% (23/41) in patients with persistent AF and 32.2% (19/59) in patients with paroxysmal AF. Thus, based on the above discussion, it is easy to understand that patient-related characteristics of persistent AF and CHA_2_DS_2_-VASc score were identified as the most relevant risk factors for SCE during AF ablation procedure.

In the present study, 106 lesions in 42 patients were identified by hDWI after ablation, but none of the patients exhibited clinical symptoms. Meanwhile, no significant cognitive decline was detected in SCE patients by the MoCA test during the 3-month follow-up. Of the 106 lesions identified, 53 (50%) were small (< 3 mm), 52 (49%) were medium (3 mm ≤ diameter < 10 mm), and only one (1%) was large (≥ 10 mm). In a study conducted by Dr. Deneke et al. [42], 50 acute lesions in 14 AF patients were identified by post-ablation cerebral MRI. Similar to the present study, up to 94% of the acute cerebral lesions were small or medium in size, while only 6% had a diameter greater than 10 mm [42]. Another study by Dr. Zheng et al. [33] also showed that more than 90% of the acute cerebral lesions after AF ablation were small to medium in size (< 10 mm). Meanwhile, previous studies have shown that all small- and medium-sized acute lesions disappeared during the 2–4-week MRI follow-up, while only the large acute lesions with diameters greater than 10 mm developed into the chronic cerebral infarcts [30, 31, 42, 43]. Consistent with the present study, Dr. Deneke et al. [42] and Dr. Haeusler et al. [43] also reported no association between the occurrence of SCE after AF ablation and cognitive impairment. To date, no AF ablation study has found a direct association between post-ablation SCE and adverse neuropsychological outcomes, although Dr. Gaita et al. [44] reported that patients with paroxysmal and persistent AF had a higher prevalence of SCE and poorer cognitive performance than subjects in sinus rhythm.

### Study limitations

The present study also has several limitations. First, the 3-month follow-up did not incorporate cerebral hDWI to assess the prognosis of acute SCE. As previously discussed, several studies have shown that nearly 90% of the acute SCEs disappeared during the 2–4 week MRI follow-up [30, 31, 42, 43]. While the impact of acute SCE on cognitive function was correspondingly evaluated during the follow-up period, the 3-month duration might be too short to identify cognitive decline in SCE patients. However, the primary aim of this study was to compare the periprocedural incidence of SCE in AF patients undergoing catheter ablation with the HPSD strategy versus the conventional approach, a question that was precisely addressed in the current study by performing pre-procedural and post-procedural 3.0 T cerebral high-resolution DWI. Second, the patient cohort in the current study is different from real-world AF ablation cohorts. To reduce the risk of atrial arrhythmia recurrence after AF ablation and ensure participant homogeneity, we excluded the patients with notable atrial cardiomyopathy, such as those with moderate to severe valvular heart disease or a significantly enlarged left atrium (left atrial diameter ≥ 55 mm). Third, the sample size of the present study was determined based on the optimistic hypothesis that the incidence of SCE would be 60% in the conventional group and 30% in the HPSD group. Consequently, the negative findings of this study may be attributed to the limited sample size of 100 patients, which might not have provided sufficient power to detect differences in SCE between the HPSD group and the conventional group. However, the present study does not reveal any potential trend in the incidence of SCE between the two groups. If considering the possibility that the current study’s negative findings are merely due to the limited sample size of 100 patients determined by the optimistic hypothesis, another larger randomized study is planned to be designed. With the current data indicating 40% SCE incidence in the conventional group and 44% in the HPSD group, the future larger randomized study would need to enroll at least 5614 patients to ensure 80% power to detect SCE differences between the two groups, with the potential to overturn the current negative result, assuming a consistent maximum 15% drop-out rate at an *α* level of 0.05. Meanwhile, no significant differences or trends were observed between the two groups with regard to the average number of lesions, maximum lesion diameter, and total lesion volume per patient. Thus, the likelihood that the current negative findings are mainly due to the small sample size determined by the optimistic hypothesis is extremely low. To further verify these findings of the present study, future large-scale, multi-center, randomized studies are warranted. Finally, as described in the current study, the HPSD strategy was performed using the STSF catheter with a power setting of 50 W, not the “very HPSD strategy” that utilizes the QDOT catheter with a power setting of 90 W for 4 S. Therefore, the conclusions of the present study cannot be extended to the “very HPSD strategy” using the QDOT catheter.

## Conclusions

Involving a small sample size of 100 AF patients and utilizing the high-resolution cerebral DWI technique, the present Reduce-IT study reveals a similar periprocedural incidence of SCE between AF patients undergoing catheter ablation with the HPSD strategy using the STSF catheter and those employing the conventional approach with the ST catheter. Meanwhile, lesion metrics, including average lesion count, maximum lesion diameter, and total lesion volume, also exhibited no significant differences between the two groups. To further verify these findings, future large-scale, multi-center, randomized studies are warranted.

### Supplementary Information

**Additional file 1: Table S1.** The oral anticoagulants information of enrolled AF patients.

## Data Availability

All the analyzed data are included in this published article. The datasets are available from the corresponding author on reasonable request.

## Abbreviations

ACT: Activated clotting time
ADC: Apparent diffusion coefficient
AF: Atrial fibrillation
BMI: Body mass index
DWI: Diffusion-weighted magnetic resonance imaging
hDWI: High-resolution diffusion-weighted magnetic resonance imaging
HPSD: High-power short-duration
IQR: Interquartile range
LAD: Left atrial diameter
LVEF: Left ventricular ejection fraction
MoCA: Montreal Cognitive Assessment
MRI: Magnetic resonance imaging
PVI: Pulmonary vein isolation
RF: Radiofrequency
SCE: Silent cerebral embolism
ST: Smart Touch
STSF: Smart Touch Surround Flow
TIA: Transient ischemic attack
